# Are we failing to “build on the scientific basis of medicine?”

**Published:** 2018-07-27

**Authors:** Sylvain Coderre, Ira Ripstein, Pamela Veale, Kevin McLaughlin

**Affiliations:** 1 Office of Undergraduate Medical Education, Cumming School of Medicine, University of Calgary, Alberta, Canada; 2 Office of Undergraduate Medical Education, Max Rady College of Medicine, University of Manitoba, Manitoba, Canada

## Abstract

In this article, we question if and why the Canadian national medical education organizations have failed to introduce or promote changes that compel or encourage Canadian medical schools to heed the recommendation from the Future of Medical Education in Canada report to “build on the scientific basis of medicine.” We end by offering suggestions on how these organizations could help Canadian medical schools build in the scientific basis of medicine.

“An investment in knowledge pays the best interest.”

- Benjamin Franklin

In 2010, the Association of Faculties of Medicine of Canada (AFMC) released the Future of Medical Education in Canada (FMEC) project report that included “a collective vision for MD education” articulated in the form of ten recommendations: address individual and community needs; enhance admissions processes; build on the scientific basis of medicine; promote prevention and public health; address the hidden curriculum; diversify learning contexts; value generalism; advance intra- and inter-professionalism practice; adopt and competency-based and flexible approach; and foster medical leadership.^1^ Appropriately, most of the stakeholder organizations involved in medical education in Canada, including the AFMC, the Committee on Accreditation of Canadian Medical Schools (CACMS), the Medical Council of Canada (MCC), and the Canadian Association for Medical Education (CAME) have, to varying degrees, embraced these recommendations. However, we feel that the recommendation to “build on the scientific basis of medicine” has been relatively neglected.

## Which of the FMEC project recommendations have garnered the most attention?

There is no valid, direct measure of partisanship within the medical education community, but we can study indirectly where attention has been directed since the publication of the FMEC project recommendations in 2010. Two ways of doing this are to examine changes within the national medical education organizations since the publication of the FMEC project recommendations and by quantifying publications in the medical education literature on the themes of the FMEC recommendations.

Since 2010, the AFMC/CAME has chosen several of the FMEC recommendations as their themes for the annual Canadian Conference on Medical Education (CCME), such as leadership, social accountability, admissions, and diversifying learning context. CACMS has introduced new accreditation standards that map to many of the FMEC recommendations, including community needs, generalism, inter-professional education, leadership, and diversification of learning contexts.^2,3^ As for the Medical Council of Canada, they completed a “Blueprint Project” focused on health promotion, illness prevention, and chronic disease management, in addition to increased emphasis on communication with colleagues and other healthcare professionals.^4,5^

**Figure 1:**
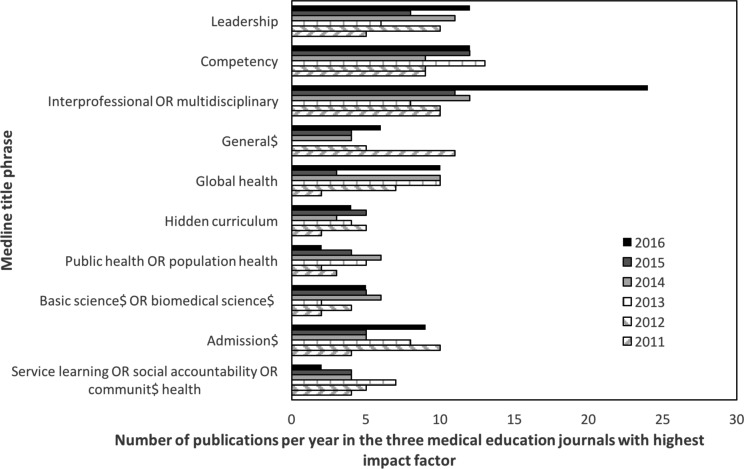
Number of publications in the three medical education journals with highest impact factor between 2011 and 2016

To compare the number of publications on each FMEC recommendation, we conducted a literature search of MEDLINE^®^ database from January 1, 2011 to December 31, 2016 for articles published in the three medical education journals with the highest impact factors (Academic Medicine, Medical Education, and Advances in Health Science Education).^6^ The search terms that we used for each theme were the following title phrases: “service learning OR social accountability OR communit$ health;” “admission$;” “basic science$ OR biomedical science$;” “public health OR population health;” “hidden curriculum;” “global health;” “general$;” “interprofessional OR inderdisciplinary;” “competency;” and “leadership.” The number of publications per year since 2010 is shown in Figure 1.

While our approach to comparing the relative attention given to the FMEC recommendations lacks scientific rigour and our data should be considered, at best, semi-quantitative – they clearly suggest that since 2010, the medical education community has focused more on promoting issues such as inter-professional education, generalism, leadership, and competency-based medical education than matters related to the biomedical sciences.

## Have we built on the scientific basis of medicine?

If the intention of the FMEC MD 2015 report was to highlight “Five years of innovations at Canadian medical schools” on the 10 FMEC recommendation, it is telling that regarding the recommendation to build on the scientific basis for medicine there was a call for “…leadership from AFMC on this recommendation…” and, specifically, for the creation of “…a national forum to discuss how and where the sciences foundational to the practice of medicine are best taught.”^7^ Canadian graduates do not have formal requirement to demonstrate mastery of the basic sciences as part of their licensure requirements, and based upon the Blueprint Project, the basic science content for future versions of the part I and II exams will be unchanged. With regards to accreditation, there is a longstanding expectation that “…The faculty of a medical school ensure that the medical curriculum includes content from the biomedical, behavioral, and socioeconomic sciences to support medical students’ mastery of contemporary scientific knowledge and concepts and the methods fundamental to applying them to the health of individuals and populations.”^2^ While one could argue this accreditation standard for the biomedical sciences content secures its place in the undergraduate curriculum, the purpose of the FMEC project recommendation to build on the scientific basis of medicine was to shape change within the medical education curriculum so that there is an increased focus on the biomedical sciences. As currently stated, we feel that the bar for this standard is very low and it is hard to imagine any medical school struggling to meet this. The wording “…includes content from…” implies that a dash of anatomy and a pinch of pathology and physiology should suffice for the purposes of accreditation, and by suggesting that the biomedical sciences play a supporting role, we can infer that there is no actual requirement for students to demonstrate mastery of the basic sciences.”^2^

## Why have we failed to build on the scientific basis of medicine?

There are good data to suggest that the biomedical sciences provide a body of knowledge that is foundational to medical education and clinical practice, that biomedical knowledge and clinical knowledge are inter-related, and that interventions designed to improve biomedical knowledge can improve clinical performance.^8-10^ So why are we failing to build on this scientific basis? We would offer three explanations: 1) there may be a perception that we have already met this FMEC recommendation since all fully accredited medical schools are deemed to have sufficient biomedical science content; 2) the recommendation to build on the scientific basis of medicine may lack the allure of other recommendations, such as advancing inter-professionalism practice; and 3) the activities of the national medical education organizations may have introduced a performance bias by making ten concurrent [unweighted] recommendations and then providing additional resources and/or external motivation for only some of these.

## How we can build on the scientific basis of medicine

Given the repeated recommendation in FMEC MD 2015 to build on the scientific basis of medicine^9^ (in addition to similar recommendations from other organizations in medical education),^11^ rather than debating the merits of this recommendation, we need to move the discussion to implementation. We feel that this requires action by the national medical education organizations, and for each of these we have a suggestion:

1. The AFMC/CAME could provide leadership and consider making this as the theme of a future CCME conference (CCME 2019: “Investing in knowledge”).
2. Similar to the situation in the United States,^12^ the MCC could consider making mastery of biomedical science a requirement for licensure in Canada (or at least increase the biomedical science content of the existing exams).
3. Rather than CACMS accrediting a curriculum that vaguely “…includes content from…” biomedical sciences, this organization could define the minimum biomedical content that all undergraduate curricula need to follow and also consider making it a requirement that students demonstrate mastery of biomedical content.^2^
